# Opipramol dipicrate

**DOI:** 10.1107/S1600536810026565

**Published:** 2010-07-10

**Authors:** Jerry P. Jasinski, Albert E. Pek, B. P. Siddaraju, H. S. Yathirajan, B. Narayana

**Affiliations:** a Department of Chemistry, Keene State College, 229 Main Street, Keene, NH 03435-2001, USA; b Department of Chemistry, V. V. Puram College of Science, Bangalore 560 004, India; c Department of Studies in Chemistry, University of Mysore, Manasagangotri, Mysore 570 006, India; d Department of Studies in Chemistry, Mangalore University, Mangalagangotri 574 199, India

## Abstract

In the crystal structure of the title compound, C_23_H_31_N_3_O^2+^·2C_6_H_2_N_3_O_7_^−^, {systematic name: 1-[3-(5*H*-dibenz[*b*,*f*]azepin-5-yl)prop­yl]-4-(2-hy­droxy­eth­yl)piperazine-1,4-diium bis­(2,4,6-trinitro­phrenolate)} the piperazine group in the opipramol dication is protonated at both N atoms. Each picrate anion inter­acts with the protonated N atom in the cation through a bifurcated N—H⋯O hydrogen bond, forming an *R*_2_^1^(6) ring motif. In the cation, the dihedral angle between the mean planes of the two benzene rings is 50.81 (8) Å. Inter­molecular O—H⋯O and weak C—H⋯O hydrogen bonds, and weak π-ring and π–π stacking inter­actions dominate the crystal packing.

## Related literature

For the use of opipramol in the treatment of anxiety disorder, see: Moller *et al.* (2001 ▶). For its use in the preparation of amine derivatives, see: Shriner *et al.* (1980 ▶). For crystal engineering research, see: Desiraju *et al.* (1989 ▶). For related structures, see: Bindya *et al.* (2007 ▶); Jasinski *et al.* (2010 ▶); Yathirajan *et al.* (2007 ▶). For bond-length data, see: Allen *et al.* (1987 ▶).
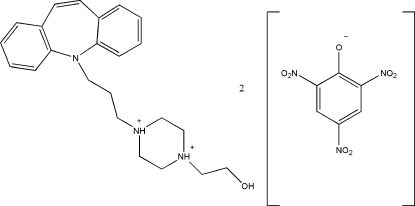


## Experimental

### 

#### Crystal data

C_23_H_31_N_3_O^2+^·2C_6_H_2_N_3_O_7_^−^
*M* *_r_* = 821.72
Triclinic, 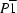

*a* = 7.3838 (8) Å
*b* = 12.0400 (13) Å
*c* = 22.074 (2) Å
α = 74.821 (1)°
β = 84.355 (2)°
γ = 73.866 (2)°
*V* = 1818.6 (3) Å^3^
*Z* = 2
Mo *K*α radiation
μ = 0.12 mm^−1^
*T* = 100 K
0.55 × 0.50 × 0.14 mm

#### Data collection

Bruker APEXII CCD diffractometer
Absorption correction: multi-scan (*SADABS*; Bruker, 2008 ▶) *T*_min_ = 0.937, *T*_max_ = 0.983
10692 measured reflections
10692 independent reflections
7831 reflections with *I* > 2σ(*I*)

#### Refinement

*R*[*F* ^2^ > 2σ(*F* ^2^)] = 0.049
*wR*(*F* ^2^) = 0.123
*S* = 0.98
10692 reflections
669 parameters
H atoms treated by a mixture of independent and constrained refinement
Δρ_max_ = 0.64 e Å^−3^
Δρ_min_ = −0.39 e Å^−3^

### 

Data collection: *APEX2* (Bruker, 2008 ▶); cell refinement: *SAINT* (Bruker, 2008 ▶); data reduction: *SAINT*; program(s) used to solve structure: *SHELXS97* (Sheldrick, 2008 ▶); program(s) used to refine structure: *SHELXTL* (Sheldrick, 2008 ▶); molecular graphics: *SHELXTL*; software used to prepare material for publication: *SHELXTL*.

## Supplementary Material

Crystal structure: contains datablocks global, I. DOI: 10.1107/S1600536810026565/xu2786sup1.cif

Structure factors: contains datablocks I. DOI: 10.1107/S1600536810026565/xu2786Isup2.hkl

Additional supplementary materials:  crystallographic information; 3D view; checkCIF report

## Figures and Tables

**Table 1:** Hydrogen-bond geometry (Å, °)

*D*—H⋯*A*	*D*—H	H⋯*A*	*D*⋯*A*	*D*—H⋯*A*
N1—H1⋯O1*B*^i^	0.91 (2)	1.85 (2)	2.6901 (16)	152.6 (18)
N1—H1⋯O7*B*^i^	0.91 (2)	2.383 (19)	3.0466 (17)	130.0 (16)
N2—H2⋯O1*A*^ii^	0.90 (2)	1.78 (2)	2.6204 (16)	154.6 (19)
N2—H2⋯O2*A*^ii^	0.90 (2)	2.43 (2)	3.0711 (16)	128.2 (16)
O1—H1*C*⋯O1*B*^i^	0.82	2.50	3.1600 (19)	138
O1—H1*C*⋯O7*B*^i^	0.82	2.38	3.0841 (18)	144

Symmetry codes: (i) 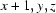; (ii) 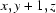.

**Table 2:** *Y*—*X*⋯*Cg* π ring inter­actions (Å) *Cg*3 and *Cg*9 are the centroids of the C10–C15 and C1*A*–C6*A* rings, respectively. *CgX*⋯Perp and *CgY*⋯Perp are the perpendicular distances between atoms *X* and *Y* and the ring centroid.

*Y*—*X*⋯*Cg*	*X*⋯*Cg*	*Y*⋯*Cg*	*X*⋯Perp
C1*A*—O1*A*⋯*Cg*3^i^	3.5674 (13)	3.6471 (17)	3.494
N3*A*—O4*A*⋯*Cg*9	3.8172 (17)	3.8173 (17)	−3.357
N3*B*—O4*B*⋯*Cg*9^ii^	3.4320 (15)	3.9391 (15)	3.288

Symmetry codes: (i) *x*, −1 + *y*, *z*; (ii) 1 − *x*, −*y*, 1 − *z*.

**Table 3:** *Cg*⋯*Cg* π stacking inter­actions (Å) *Cg*2, *Cg*3, *Cg*8 and *Cg*9 are the centroids of the C10–C15, C18–C23, C1*A*–C6*A* and C1*B*–C6*B* rings, respectively. *CgX*⋯Perp and *CgY*⋯Perp are the perpendicular distances between the ring centroid and the other ring.

	*CgX*⋯*CgY*	*CgX*⋯Perp	*CgY*⋯Perp
*Cg*2⋯*Cg*2^i^	3.8038 (11)	−3.5589 (7)	−3.5590 (7)
*Cg*3⋯*Cg*3^i^	3.7164 (10)	−3.6624 (7)	−3.6623 (7)
*Cg*8⋯*Cg*9	3.9558 (10)	−3.2475 (6)	3.3731 (6)

Symmetry code: (i) 2 − *x*, 1 − *y*, −*z*.

**Fig. 1.**
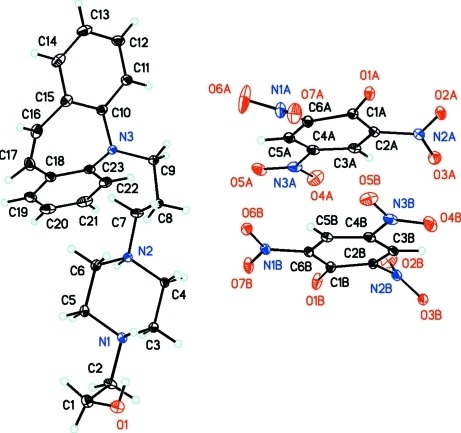
Molecular structure of, C23H33N3O+, (C6H2N3O7-)2, showing the atom labeling scheme and 30% probability displacement ellipsoids.

**Fig. 2.**
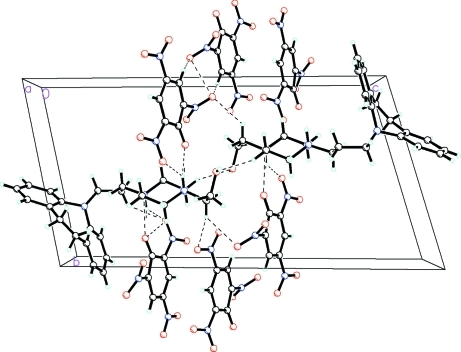
Diagram for the R21(6)···ab..graph-set motif in the cation of the title compound, C23H33N3O+, (C6H2N3O7-)2.

**Fig. 3.**
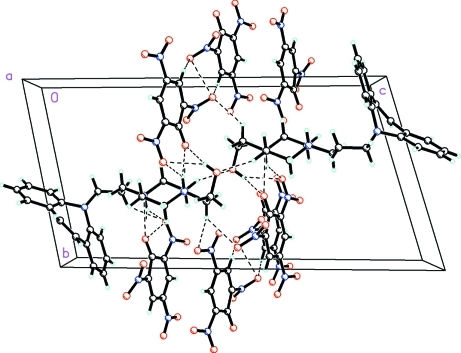
Packing diagram of the title compound viewed down the a axis. Dashed lines indicate intermolecular N—H···O and C—H···O hydrogen bond interactions.

## supplementary crystallographic information

### Comment

Opipramol (systematic IUPAC name: 4-[3-(5*H*-dibenz[b,f]azepin-5-yl)propyl]-1-piperazinethanol) is an antidepressant and anxiolytic typically used in the treatment of generalized anxiety disorder (Moller *et al.*, 2001). Opipramol, a drug widely prescribed in Germany, is a tricyclic compound with no reuptake-inhibiting properties. However, it has pronounced D2-, 5-HT2-, and H1-blocking potential and high affinity to sigma receptors (sigma-1 and sigma-2). Crystalline picrates have commonly been used in the preparation of amine derivatives in qualitative organic chemistry (Shriner *et al.*, 1980). Hydrogen bonding plays a key role in molecular recognition and crystal engineering research (Desiraju *et al.*, 1989). The crystal structures of trifluoperazinium dipicrate (Yathirajan *et al.*, 2007), amitriptylinium picrate (Bindya *et al.*, 2007) and imatinibium dipicrate (Jasinski *et al.*, 2010) have been reported. The present work reports the crystal structure of the salt formed by the interaction between 4-[3-(5*H*-dibenz[b,f]azepin-5-yl)propyl]-1-piperazinethanol dihydrochloride and 2,4,6-trinitrophenol in aqueous medium.

In opipramol dipicrate, C_23_H_33_N_3_O^+^, (C_6_H_2_N_3_O_7_^-^)_2_, the piperazine group in the opipramol cation is protonated at both of the N atoms. The 6-membered piperazine group (N1/C5/C6/N2/C4/C3) adopts a slightly distorted chair conformation with puckering parameters Q, θ and φ of 0.584 (7)A%, 178.40°, and 312.658 (8)°, respectively (Fig.1). For an ideal chair θ has a value of 0 or 180°. Bond distances and angles are in normal ranges (Allen *et al.*,  1987).  *R*_2_^1^(6) graph-set motifs are formed between piperazine N1—H1 and N2—H2 groups and the picrate anions (labeled A and B) through bifurcated N—H···O hydrgen bonds (Fig. 2). The mean plane of the two *o*-NO_2_ groups in the two picrate anions are twisted by 31.8 (8)°, 31.8 (8)° in both the A ring B rings with respect to the mean planes of the 6-membered benzene rings. The *p*-NO_2_ groups in both picrate anions are nearly in the plane of the ring (torsion angles O4A—N3A—C4A—C3A = -1.7 (2)°; O4B—N3B—C4B—C3B = -12.1 (2)°). An extensive array of weak O—H···O and C—H···O intermolecular hydrogen bonds (Table 1), weak π-ring (Table 2) and π-π (Table 3) stacking interactions dominate crystal packing in the unit cell (Fig. 3).

### Experimental

Opipramol dihydrochloride (4.38 g, 0.01 mol) was dissolved in 25 ml of water and picric acid (2.4 g, 0.01 mol) was dissolved in 25 ml of water. Both the solutions were mixed and stirred in a beaker at room temperature for one hour. The mixture was kept aside for two days at room temperature. The formed salt was filtered & dried in vacuum desiccator over phosphorous pentoxide. The salt was recrystallized from DMSO by slow evaporation (m.p: 453–455 K).

### Refinement

The H1C, H1 and H2 atoms were located by a Fourier map. These H atoms and the rest of the H atoms were then positioned geometrically and allowed to ride on their parent atoms with Atom—H lengths of 0.82Å (O1), 0.91Å (NH), 0.93 Å (CH), 0.97Å (CH_2_) or (CH_3_). Isotropic displacement parameters for these atoms were set to 1.40 times (OH), 1.20 times (NH), 1.20 (CH) or 1.22 (CH_2_) times (CH_3_) *U*_eq_ of the parent atom.  The highest and lowest peaks (0.64 & 0.31 eÅ^-3^) are located 1.21Å and 0.31Å from atoms N1A and H1C, respectively.

### Crystal data

**Table:**

C_23_H_31_N_3_O^2+^·2C_6_H_2_N_3_O_7_^−^	*Z* = 2
*M**_r_* = 821.72	*F*(000) = 856
Triclinic, *P*1	*D*_x_ = 1.501 Mg m^−^^3^
Hall symbol: -p 1	Mo *K*α radiation, λ = 0.71073 Å
*a* = 7.3838 (8) Å	Cell parameters from 5178 reflections
*b* = 12.0400 (13) Å	θ = 2.9–30.4°
*c* = 22.074 (2) Å	µ = 0.12 mm^−^^1^
α = 74.821 (1)°	*T* = 100 K
β = 84.355 (2)°	Plate, yellow
γ = 73.866 (2)°	0.55 × 0.50 × 0.14 mm
*V* = 1818.6 (3) Å^3^	

### Data collection

**Table:**

Bruker APEXII CCD diffractometer	10692 independent reflections
Radiation source: fine-focus sealed tube	7831 reflections with *I* > 2σ(*I*)
graphite	*R*_int_ = 0.0000
ω scans	θ_max_ = 31.1°, θ_min_ = 1.8°
Absorption correction: multi-scan (*SADABS*; Bruker, 2008)	*h* = −10→10
*T*_min_ = 0.937, *T*_max_ = 0.983	*k* = −16→17
10692 measured reflections	*l* = 0→30

### Refinement

**Table:**

Refinement on *F*^2^	Primary atom site location: structure-invariant direct methods
Least-squares matrix: full	Secondary atom site location: difference Fourier map
*R*[*F*^2^ > 2σ(*F*^2^)] = 0.049	Hydrogen site location: inferred from neighbouring sites
*wR*(*F*^2^) = 0.123	H atoms treated by a mixture of independent and constrained refinement
*S* = 0.98	*w* = 1/[σ^2^(*F*_o_^2^) + (0.0431*P*)^2^ + 1.3196*P*] where *P* = (*F*_o_^2^ + 2*F*_c_^2^)/3
10692 reflections	(Δ/σ)_max_ < 0.001
669 parameters	Δρ_max_ = 0.64 e Å^−^^3^
0 restraints	Δρ_min_ = −0.39 e Å^−^^3^

### Special details

**Table:**

Geometry. All esds (except the esd in the dihedral angle between two l.s. planes) are estimated using the full covariance matrix. The cell esds are taken into account individually in the estimation of esds in distances, angles and torsion angles; correlations between esds in cell parameters are only used when they are defined by crystal symmetry. An approximate (isotropic) treatment of cell esds is used for estimating esds involving l.s. planes.
Refinement. Refinement of *F*^2^ against ALL reflections. The weighted *R*-factor *wR* and goodness of fit *S* are based on *F*^2^, conventional *R*-factors *R* are based on *F*, with *F* set to zero for negative *F*^2^. The threshold expression of *F*^2^ > σ(*F*^2^) is used only for calculating *R*-factors(gt) *etc*. and is not relevant to the choice of reflections for refinement. *R*-factors based on *F*^2^ are statistically about twice as large as those based on *F*, and *R*- factors based on ALL data will be even larger.

### Fractional atomic coordinates and isotropic or equivalent isotropic displacement parameters (Å^2^)

**Table:**

	*x*	*y*	*z*	*U*_iso_*/*U*_eq_	
N1	1.02455 (17)	0.60675 (11)	0.37838 (6)	0.0151 (2)	
N1A	0.8758 (2)	0.06526 (12)	0.19787 (7)	0.0288 (3)	
N1B	0.49985 (18)	0.35440 (11)	0.34309 (6)	0.0191 (2)	
N2	0.83911 (17)	0.63062 (11)	0.26315 (5)	0.0154 (2)	
N2A	0.36714 (18)	−0.11954 (11)	0.30334 (6)	0.0201 (3)	
N2B	0.0123 (2)	0.16475 (12)	0.46259 (7)	0.0240 (3)	
N3	0.65920 (18)	0.69770 (11)	0.08383 (6)	0.0183 (2)	
N3A	0.2539 (2)	0.30858 (12)	0.23707 (7)	0.0296 (3)	
N3B	0.6228 (2)	−0.07470 (12)	0.40742 (6)	0.0262 (3)	
O1	1.32600 (16)	0.50804 (10)	0.47765 (5)	0.0238 (2)	
H1C	1.3355	0.4665	0.4528	0.036*	
O1A	0.73234 (15)	−0.13584 (9)	0.23643 (5)	0.0225 (2)	
O1B	0.13483 (17)	0.36702 (10)	0.41214 (6)	0.0335 (3)	
O2A	0.45142 (16)	−0.22069 (9)	0.29802 (6)	0.0243 (2)	
O2B	−0.12159 (18)	0.22565 (14)	0.43026 (7)	0.0439 (4)	
O3A	0.23060 (17)	−0.10226 (11)	0.33974 (6)	0.0309 (3)	
O3B	−0.00152 (19)	0.10754 (10)	0.51708 (6)	0.0302 (3)	
O4A	0.0937 (2)	0.31534 (12)	0.25984 (7)	0.0451 (4)	
O4B	0.5803 (2)	−0.16385 (10)	0.44083 (6)	0.0363 (3)	
O5A	0.3147 (2)	0.39677 (10)	0.21386 (6)	0.0366 (3)	
O5B	0.76309 (19)	−0.07890 (11)	0.37238 (6)	0.0350 (3)	
O6A	0.9082 (2)	0.14690 (14)	0.15701 (10)	0.0721 (6)	
O6B	0.64141 (15)	0.33702 (10)	0.30921 (5)	0.0240 (2)	
O7A	0.99987 (19)	−0.02019 (13)	0.22406 (7)	0.0419 (3)	
O7B	0.40979 (18)	0.45584 (10)	0.34673 (6)	0.0327 (3)	
C1	1.2578 (2)	0.63008 (14)	0.44618 (8)	0.0226 (3)	
C1A	0.6250 (2)	−0.03839 (12)	0.24106 (7)	0.0169 (3)	
C1B	0.2478 (2)	0.27017 (12)	0.40804 (7)	0.0188 (3)	
C2	1.0532 (2)	0.65724 (14)	0.43134 (7)	0.0201 (3)	
C2A	0.4357 (2)	−0.01908 (12)	0.26842 (7)	0.0174 (3)	
C2B	0.2017 (2)	0.15826 (13)	0.43494 (7)	0.0180 (3)	
C3	0.8236 (2)	0.60313 (13)	0.37804 (7)	0.0158 (3)	
C3A	0.3154 (2)	0.09238 (13)	0.26613 (7)	0.0203 (3)	
C3B	0.3198 (2)	0.04765 (13)	0.43780 (7)	0.0203 (3)	
C4	0.7918 (2)	0.55480 (12)	0.32445 (6)	0.0157 (3)	
C4A	0.3782 (2)	0.19229 (13)	0.23820 (7)	0.0223 (3)	
C4B	0.4978 (2)	0.04110 (12)	0.40896 (7)	0.0196 (3)	
C5	1.0750 (2)	0.68021 (13)	0.31640 (7)	0.0173 (3)	
C5A	0.5629 (2)	0.18292 (13)	0.21535 (7)	0.0224 (3)	
C5B	0.5537 (2)	0.14175 (13)	0.37891 (7)	0.0180 (3)	
C6	1.0402 (2)	0.63363 (13)	0.26278 (7)	0.0181 (3)	
C6A	0.6808 (2)	0.07208 (13)	0.21871 (7)	0.0202 (3)	
C6B	0.4335 (2)	0.25296 (12)	0.37819 (7)	0.0170 (3)	
C7	0.8042 (2)	0.58956 (14)	0.20786 (7)	0.0208 (3)	
C8	0.5958 (2)	0.61594 (14)	0.19562 (7)	0.0199 (3)	
C9	0.5705 (2)	0.61345 (14)	0.12799 (7)	0.0217 (3)	
C10	0.7251 (2)	0.67135 (13)	0.02498 (7)	0.0196 (3)	
C11	0.6288 (3)	0.61785 (14)	−0.00504 (7)	0.0252 (3)	
C12	0.7035 (3)	0.58299 (15)	−0.05966 (8)	0.0309 (4)	
C13	0.8755 (3)	0.60076 (16)	−0.08394 (8)	0.0327 (4)	
C14	0.9697 (3)	0.65574 (15)	−0.05503 (8)	0.0296 (4)	
C15	0.8954 (2)	0.69466 (14)	−0.00106 (7)	0.0229 (3)	
C16	0.9953 (2)	0.75994 (16)	0.02502 (8)	0.0280 (3)	
C17	0.9182 (3)	0.84618 (16)	0.05467 (8)	0.0284 (4)	
C18	0.7192 (2)	0.89186 (14)	0.07048 (7)	0.0228 (3)	
C19	0.6521 (3)	1.01141 (15)	0.07358 (8)	0.0312 (4)	
C20	0.4661 (3)	1.05952 (16)	0.08865 (8)	0.0359 (4)	
C21	0.3421 (3)	0.98862 (17)	0.10155 (8)	0.0345 (4)	
C22	0.4037 (2)	0.87034 (16)	0.09885 (7)	0.0264 (3)	
C23	0.5914 (2)	0.82097 (13)	0.08345 (7)	0.0198 (3)	
H1	1.096 (3)	0.5306 (18)	0.3834 (9)	0.027 (5)*	
H2	0.769 (3)	0.7069 (19)	0.2581 (9)	0.032 (5)*	
H1A	1.334 (3)	0.6519 (17)	0.4086 (9)	0.029 (5)*	
H1B	1.265 (3)	0.6790 (17)	0.4736 (9)	0.028 (5)*	
H2A	1.000 (3)	0.7399 (18)	0.4188 (9)	0.028 (5)*	
H2B	0.984 (2)	0.6202 (16)	0.4667 (9)	0.019 (4)*	
H3A	0.748 (2)	0.6818 (15)	0.3746 (8)	0.014 (4)*	
H3B	0.797 (2)	0.5526 (16)	0.4172 (8)	0.018 (4)*	
H3C	0.192 (3)	0.0976 (17)	0.2851 (9)	0.027 (5)*	
H3D	0.280 (3)	−0.0219 (17)	0.4569 (9)	0.025 (5)*	
H4A	0.871 (2)	0.4727 (15)	0.3266 (8)	0.014 (4)*	
H4B	0.660 (3)	0.5557 (15)	0.3244 (8)	0.018 (4)*	
H5A	1.205 (3)	0.6769 (16)	0.3158 (8)	0.020 (4)*	
H5B	0.995 (3)	0.7623 (16)	0.3130 (8)	0.022 (5)*	
H5C	0.608 (3)	0.2514 (18)	0.1990 (9)	0.031 (5)*	
H5D	0.670 (3)	0.1386 (17)	0.3592 (9)	0.029 (5)*	
H6A	1.064 (3)	0.6881 (17)	0.2241 (9)	0.028 (5)*	
H6B	1.119 (3)	0.5537 (17)	0.2650 (8)	0.023 (5)*	
H7A	0.868 (3)	0.5037 (18)	0.2157 (9)	0.028 (5)*	
H7B	0.865 (3)	0.6356 (16)	0.1722 (9)	0.021 (4)*	
H8A	0.530 (2)	0.6955 (15)	0.2024 (8)	0.015 (4)*	
H8B	0.537 (2)	0.5596 (16)	0.2237 (8)	0.019 (4)*	
H9A	0.637 (3)	0.5335 (17)	0.1205 (8)	0.023 (5)*	
H9B	0.430 (3)	0.6262 (17)	0.1206 (9)	0.029 (5)*	
H11	0.513 (3)	0.6024 (18)	0.0116 (10)	0.033 (5)*	
H12	0.632 (3)	0.5471 (19)	−0.0806 (10)	0.038 (6)*	
H13	0.932 (3)	0.5741 (19)	−0.1207 (10)	0.038 (6)*	
H14	1.085 (3)	0.6724 (17)	−0.0727 (9)	0.030 (5)*	
H16	1.129 (3)	0.746 (2)	0.0137 (10)	0.044 (6)*	
H17	0.998 (3)	0.8897 (18)	0.0633 (10)	0.036 (6)*	
H19	0.740 (3)	1.0580 (18)	0.0661 (10)	0.034 (5)*	
H20	0.426 (3)	1.1418 (19)	0.0917 (10)	0.040 (6)*	
H21	0.213 (3)	1.023 (2)	0.1115 (11)	0.045 (6)*	
H22	0.313 (3)	0.8197 (17)	0.1082 (9)	0.029 (5)*	

### Atomic displacement parameters (Å^2^)

**Table:**

	*U*^11^	*U*^22^	*U*^33^	*U*^12^	*U*^13^	*U*^23^
N1	0.0133 (6)	0.0123 (5)	0.0194 (6)	−0.0018 (4)	−0.0006 (4)	−0.0052 (4)
N1A	0.0266 (7)	0.0211 (6)	0.0364 (8)	−0.0088 (6)	0.0042 (6)	−0.0022 (6)
N1B	0.0171 (6)	0.0173 (6)	0.0237 (6)	−0.0042 (5)	0.0011 (5)	−0.0073 (5)
N2	0.0164 (6)	0.0132 (5)	0.0158 (5)	−0.0026 (5)	−0.0005 (4)	−0.0032 (4)
N2A	0.0165 (6)	0.0203 (6)	0.0246 (6)	−0.0040 (5)	−0.0010 (5)	−0.0083 (5)
N2B	0.0241 (7)	0.0248 (7)	0.0272 (7)	−0.0115 (6)	0.0024 (5)	−0.0092 (5)
N3	0.0225 (6)	0.0187 (6)	0.0144 (5)	−0.0071 (5)	0.0001 (5)	−0.0036 (5)
N3A	0.0403 (9)	0.0184 (6)	0.0229 (7)	0.0090 (6)	−0.0089 (6)	−0.0073 (5)
N3B	0.0341 (8)	0.0167 (6)	0.0246 (7)	0.0057 (6)	−0.0132 (6)	−0.0082 (5)
O1	0.0223 (6)	0.0215 (5)	0.0244 (5)	0.0004 (4)	−0.0065 (4)	−0.0047 (4)
O1A	0.0192 (5)	0.0133 (5)	0.0306 (6)	−0.0006 (4)	0.0033 (4)	−0.0030 (4)
O1B	0.0281 (6)	0.0145 (5)	0.0456 (7)	0.0017 (5)	0.0160 (5)	−0.0005 (5)
O2A	0.0216 (6)	0.0166 (5)	0.0340 (6)	−0.0045 (4)	0.0040 (5)	−0.0071 (5)
O2B	0.0188 (6)	0.0655 (10)	0.0417 (8)	−0.0087 (6)	−0.0041 (5)	−0.0047 (7)
O3A	0.0267 (6)	0.0318 (6)	0.0347 (7)	−0.0081 (5)	0.0110 (5)	−0.0126 (5)
O3B	0.0439 (7)	0.0259 (6)	0.0256 (6)	−0.0174 (5)	0.0102 (5)	−0.0098 (5)
O4A	0.0434 (8)	0.0295 (7)	0.0431 (8)	0.0164 (6)	0.0061 (6)	−0.0060 (6)
O4B	0.0476 (8)	0.0139 (5)	0.0443 (8)	−0.0005 (5)	−0.0108 (6)	−0.0065 (5)
O5A	0.0537 (9)	0.0153 (5)	0.0377 (7)	0.0023 (5)	−0.0136 (6)	−0.0090 (5)
O5B	0.0374 (7)	0.0278 (6)	0.0285 (6)	0.0135 (5)	−0.0034 (5)	−0.0101 (5)
O6A	0.0530 (10)	0.0301 (8)	0.1039 (15)	−0.0083 (7)	0.0356 (10)	0.0170 (9)
O6B	0.0191 (5)	0.0256 (6)	0.0279 (6)	−0.0064 (4)	0.0065 (4)	−0.0094 (5)
O7A	0.0241 (7)	0.0411 (8)	0.0543 (9)	−0.0092 (6)	−0.0031 (6)	0.0005 (7)
O7B	0.0311 (7)	0.0154 (5)	0.0498 (8)	−0.0068 (5)	0.0170 (6)	−0.0109 (5)
C1	0.0212 (8)	0.0202 (7)	0.0256 (8)	−0.0051 (6)	−0.0080 (6)	−0.0018 (6)
C1A	0.0175 (7)	0.0139 (6)	0.0171 (6)	−0.0010 (5)	−0.0033 (5)	−0.0022 (5)
C1B	0.0182 (7)	0.0141 (6)	0.0218 (7)	−0.0019 (5)	−0.0004 (5)	−0.0031 (5)
C2	0.0185 (7)	0.0190 (7)	0.0239 (7)	0.0002 (6)	−0.0065 (6)	−0.0103 (6)
C2A	0.0166 (7)	0.0159 (6)	0.0193 (6)	−0.0018 (5)	−0.0034 (5)	−0.0052 (5)
C2B	0.0180 (7)	0.0176 (6)	0.0186 (6)	−0.0057 (5)	−0.0017 (5)	−0.0034 (5)
C3	0.0123 (6)	0.0174 (6)	0.0173 (6)	−0.0030 (5)	0.0003 (5)	−0.0049 (5)
C3A	0.0182 (7)	0.0214 (7)	0.0198 (7)	0.0021 (6)	−0.0060 (5)	−0.0081 (6)
C3B	0.0285 (8)	0.0149 (6)	0.0187 (7)	−0.0060 (6)	−0.0052 (6)	−0.0042 (5)
C4	0.0173 (7)	0.0137 (6)	0.0165 (6)	−0.0053 (5)	−0.0012 (5)	−0.0028 (5)
C4A	0.0294 (8)	0.0146 (6)	0.0193 (7)	0.0042 (6)	−0.0072 (6)	−0.0061 (5)
C4B	0.0243 (8)	0.0128 (6)	0.0200 (7)	0.0020 (5)	−0.0073 (6)	−0.0060 (5)
C5	0.0144 (7)	0.0142 (6)	0.0222 (7)	−0.0039 (5)	−0.0003 (5)	−0.0021 (5)
C5A	0.0325 (9)	0.0144 (6)	0.0193 (7)	−0.0041 (6)	−0.0043 (6)	−0.0032 (5)
C5B	0.0162 (7)	0.0188 (7)	0.0186 (6)	0.0000 (5)	−0.0041 (5)	−0.0075 (5)
C6	0.0147 (7)	0.0178 (7)	0.0192 (7)	−0.0024 (5)	0.0007 (5)	−0.0026 (5)
C6A	0.0223 (7)	0.0162 (6)	0.0208 (7)	−0.0041 (6)	−0.0015 (6)	−0.0029 (5)
C6B	0.0176 (7)	0.0143 (6)	0.0192 (6)	−0.0036 (5)	−0.0011 (5)	−0.0044 (5)
C7	0.0246 (8)	0.0203 (7)	0.0162 (6)	0.0002 (6)	−0.0034 (6)	−0.0078 (6)
C8	0.0246 (8)	0.0210 (7)	0.0149 (6)	−0.0085 (6)	−0.0005 (5)	−0.0031 (5)
C9	0.0275 (8)	0.0239 (7)	0.0164 (6)	−0.0115 (6)	−0.0009 (6)	−0.0046 (6)
C10	0.0237 (7)	0.0167 (6)	0.0155 (6)	−0.0027 (6)	−0.0013 (5)	−0.0011 (5)
C11	0.0337 (9)	0.0235 (8)	0.0191 (7)	−0.0096 (7)	−0.0014 (6)	−0.0041 (6)
C12	0.0503 (11)	0.0242 (8)	0.0189 (7)	−0.0099 (8)	−0.0037 (7)	−0.0052 (6)
C13	0.0464 (11)	0.0259 (8)	0.0182 (7)	0.0012 (8)	0.0028 (7)	−0.0051 (6)
C14	0.0295 (9)	0.0263 (8)	0.0232 (8)	0.0008 (7)	0.0052 (7)	0.0000 (6)
C15	0.0220 (8)	0.0211 (7)	0.0200 (7)	−0.0004 (6)	−0.0022 (6)	−0.0001 (6)
C16	0.0195 (8)	0.0325 (9)	0.0283 (8)	−0.0076 (7)	−0.0019 (6)	0.0000 (7)
C17	0.0286 (9)	0.0300 (8)	0.0290 (8)	−0.0148 (7)	−0.0065 (7)	−0.0017 (7)
C18	0.0314 (8)	0.0204 (7)	0.0168 (7)	−0.0077 (6)	−0.0052 (6)	−0.0021 (6)
C19	0.0537 (12)	0.0206 (8)	0.0207 (8)	−0.0128 (8)	−0.0081 (7)	−0.0019 (6)
C20	0.0590 (13)	0.0199 (8)	0.0207 (8)	0.0056 (8)	−0.0101 (8)	−0.0046 (6)
C21	0.0375 (10)	0.0329 (9)	0.0219 (8)	0.0107 (8)	−0.0051 (7)	−0.0073 (7)
C22	0.0242 (8)	0.0307 (8)	0.0197 (7)	−0.0003 (7)	−0.0041 (6)	−0.0046 (6)
C23	0.0258 (8)	0.0186 (7)	0.0134 (6)	−0.0029 (6)	−0.0049 (5)	−0.0027 (5)

### Geometric parameters (Å, °)

**Table:**

N1—C5	1.4973 (18)	C3B—C4B	1.394 (2)
N1—C3	1.4973 (18)	C3B—H3D	0.944 (19)
N1—C2	1.5077 (18)	C4—H4A	0.992 (17)
N1—H1	0.91 (2)	C4—H4B	0.970 (18)
N1A—O6A	1.2087 (19)	C4A—C5A	1.391 (2)
N1A—O7A	1.2250 (19)	C4B—C5B	1.372 (2)
N1A—C6A	1.455 (2)	C5—C6	1.506 (2)
N1B—O6B	1.2256 (16)	C5—H5A	0.947 (19)
N1B—O7B	1.2370 (16)	C5—H5B	0.988 (18)
N1B—C6B	1.4473 (18)	C5A—C6A	1.364 (2)
N2—C4	1.4913 (18)	C5A—H5C	0.95 (2)
N2—C6	1.4944 (19)	C5B—C6B	1.383 (2)
N2—C7	1.4996 (18)	C5B—H5D	0.92 (2)
N2—H2	0.90 (2)	C6—H6A	0.97 (2)
N2A—O3A	1.2316 (16)	C6—H6B	0.968 (19)
N2A—O2A	1.2344 (16)	C7—C8	1.519 (2)
N2A—C2A	1.4451 (19)	C7—H7A	0.99 (2)
N2B—O2B	1.2189 (19)	C7—H7B	0.983 (18)
N2B—O3B	1.2298 (18)	C8—C9	1.532 (2)
N2B—C2B	1.460 (2)	C8—H8A	0.989 (17)
N3—C10	1.4250 (18)	C8—H8B	0.960 (18)
N3—C23	1.4264 (19)	C9—H9A	1.000 (18)
N3—C9	1.4609 (19)	C9—H9B	1.03 (2)
N3A—O4A	1.229 (2)	C10—C11	1.389 (2)
N3A—O5A	1.235 (2)	C10—C15	1.400 (2)
N3A—C4A	1.4408 (19)	C11—C12	1.393 (2)
N3B—O5B	1.2277 (19)	C11—H11	0.95 (2)
N3B—O4B	1.2334 (19)	C12—C13	1.379 (3)
N3B—C4B	1.4493 (19)	C12—H12	0.98 (2)
O1—C1	1.4207 (18)	C13—C14	1.377 (3)
O1—H1C	0.8200	C13—H13	0.97 (2)
O1A—C1A	1.2431 (17)	C14—C15	1.400 (2)
O1B—C1B	1.2504 (18)	C14—H14	0.95 (2)
C1—C2	1.505 (2)	C15—C16	1.462 (2)
C1—H1A	0.97 (2)	C16—C17	1.336 (3)
C1—H1B	0.96 (2)	C16—H16	0.97 (2)
C1A—C2A	1.449 (2)	C17—C18	1.461 (2)
C1A—C6A	1.450 (2)	C17—H17	0.95 (2)
C1B—C2B	1.442 (2)	C18—C23	1.403 (2)
C1B—C6B	1.447 (2)	C18—C19	1.404 (2)
C2—H2A	0.94 (2)	C19—C20	1.379 (3)
C2—H2B	0.965 (18)	C19—H19	0.95 (2)
C2A—C3A	1.379 (2)	C20—C21	1.380 (3)
C2B—C3B	1.364 (2)	C20—H20	0.97 (2)
C3—C4	1.5114 (19)	C21—C22	1.385 (3)
C3—H3A	0.944 (17)	C21—H21	0.95 (2)
C3—H3B	0.956 (18)	C22—C23	1.395 (2)
C3A—C4A	1.381 (2)	C22—H22	1.00 (2)
C3A—H3C	0.960 (19)		
			
C5—N1—C3	109.19 (11)	N1—C5—H5A	108.6 (11)
C5—N1—C2	110.78 (11)	C6—C5—H5A	108.5 (11)
C3—N1—C2	110.41 (11)	N1—C5—H5B	106.6 (10)
C5—N1—H1	109.1 (12)	C6—C5—H5B	110.4 (11)
C3—N1—H1	106.7 (12)	H5A—C5—H5B	111.4 (15)
C2—N1—H1	110.5 (12)	C6A—C5A—C4A	118.59 (14)
O6A—N1A—O7A	123.06 (16)	C6A—C5A—H5C	120.0 (12)
O6A—N1A—C6A	118.13 (15)	C4A—C5A—H5C	121.4 (12)
O7A—N1A—C6A	118.74 (13)	C4B—C5B—C6B	119.73 (14)
O6B—N1B—O7B	122.01 (13)	C4B—C5B—H5D	122.4 (12)
O6B—N1B—C6B	118.63 (12)	C6B—C5B—H5D	117.8 (12)
O7B—N1B—C6B	119.32 (12)	N2—C6—C5	111.06 (12)
C4—N2—C6	109.13 (11)	N2—C6—H6A	107.4 (12)
C4—N2—C7	112.96 (11)	C5—C6—H6A	107.9 (12)
C6—N2—C7	110.53 (11)	N2—C6—H6B	108.0 (11)
C4—N2—H2	110.9 (13)	C5—C6—H6B	111.3 (11)
C6—N2—H2	106.2 (13)	H6A—C6—H6B	111.0 (16)
C7—N2—H2	106.9 (13)	C5A—C6A—C1A	124.68 (15)
O3A—N2A—O2A	121.74 (13)	C5A—C6A—N1A	117.16 (14)
O3A—N2A—C2A	118.85 (12)	C1A—C6A—N1A	118.16 (13)
O2A—N2A—C2A	119.34 (12)	C5B—C6B—C1B	123.28 (13)
O2B—N2B—O3B	124.29 (15)	C5B—C6B—N1B	116.20 (13)
O2B—N2B—C2B	118.14 (13)	C1B—C6B—N1B	120.47 (12)
O3B—N2B—C2B	117.58 (14)	N2—C7—C8	112.82 (12)
C10—N3—C23	116.26 (11)	N2—C7—H7A	107.2 (11)
C10—N3—C9	117.10 (12)	C8—C7—H7A	112.1 (11)
C23—N3—C9	118.09 (12)	N2—C7—H7B	104.2 (11)
O4A—N3A—O5A	123.17 (14)	C8—C7—H7B	109.4 (11)
O4A—N3A—C4A	118.62 (15)	H7A—C7—H7B	110.9 (15)
O5A—N3A—C4A	118.21 (15)	C7—C8—C9	109.79 (13)
O5B—N3B—O4B	123.64 (14)	C7—C8—H8A	109.1 (10)
O5B—N3B—C4B	118.44 (14)	C9—C8—H8A	109.9 (10)
O4B—N3B—C4B	117.91 (15)	C7—C8—H8B	112.2 (11)
C1—O1—H1C	109.5	C9—C8—H8B	108.8 (11)
O1—C1—C2	110.26 (13)	H8A—C8—H8B	107.1 (14)
O1—C1—H1A	111.6 (12)	N3—C9—C8	110.31 (12)
C2—C1—H1A	111.5 (12)	N3—C9—H9A	105.3 (10)
O1—C1—H1B	109.2 (12)	C8—C9—H9A	110.6 (11)
C2—C1—H1B	106.8 (12)	N3—C9—H9B	114.5 (11)
H1A—C1—H1B	107.3 (16)	C8—C9—H9B	109.6 (11)
O1A—C1A—C2A	126.28 (13)	H9A—C9—H9B	106.4 (15)
O1A—C1A—C6A	121.87 (14)	C11—C10—C15	119.85 (14)
C2A—C1A—C6A	111.85 (12)	C11—C10—N3	121.27 (14)
O1B—C1B—C2B	121.18 (14)	C15—C10—N3	118.80 (14)
O1B—C1B—C6B	127.15 (14)	C10—C11—C12	120.62 (17)
C2B—C1B—C6B	111.66 (12)	C10—C11—H11	120.9 (12)
C1—C2—N1	112.61 (12)	C12—C11—H11	118.4 (13)
C1—C2—H2A	110.8 (12)	C13—C12—C11	119.76 (17)
N1—C2—H2A	106.4 (12)	C13—C12—H12	121.0 (12)
C1—C2—H2B	110.7 (11)	C11—C12—H12	119.2 (12)
N1—C2—H2B	104.6 (11)	C14—C13—C12	119.82 (16)
H2A—C2—H2B	111.4 (16)	C14—C13—H13	119.3 (13)
C3A—C2A—N2A	116.23 (13)	C12—C13—H13	120.9 (13)
C3A—C2A—C1A	123.64 (14)	C13—C14—C15	121.58 (17)
N2A—C2A—C1A	120.05 (12)	C13—C14—H14	120.5 (12)
C3B—C2B—C1B	125.93 (14)	C15—C14—H14	117.8 (12)
C3B—C2B—N2B	117.41 (13)	C10—C15—C14	118.24 (16)
C1B—C2B—N2B	116.65 (13)	C10—C15—C16	122.62 (14)
N1—C3—C4	111.05 (11)	C14—C15—C16	119.13 (16)
N1—C3—H3A	107.0 (10)	C17—C16—C15	126.76 (16)
C4—C3—H3A	111.2 (10)	C17—C16—H16	118.1 (13)
N1—C3—H3B	107.5 (11)	C15—C16—H16	114.5 (13)
C4—C3—H3B	109.9 (11)	C16—C17—C18	127.92 (16)
H3A—C3—H3B	110.0 (14)	C16—C17—H17	117.6 (13)
C2A—C3A—C4A	119.18 (15)	C18—C17—H17	114.2 (13)
C2A—C3A—H3C	118.5 (11)	C23—C18—C19	118.16 (16)
C4A—C3A—H3C	122.3 (11)	C23—C18—C17	122.83 (14)
C2B—C3B—C4B	117.56 (14)	C19—C18—C17	119.01 (16)
C2B—C3B—H3D	121.2 (11)	C20—C19—C18	121.74 (18)
C4B—C3B—H3D	121.2 (11)	C20—C19—H19	120.6 (13)
N2—C4—C3	110.39 (11)	C18—C19—H19	117.6 (13)
N2—C4—H4A	106.7 (10)	C19—C20—C21	119.48 (17)
C3—C4—H4A	112.7 (10)	C19—C20—H20	119.4 (13)
N2—C4—H4B	108.1 (10)	C21—C20—H20	121.1 (13)
C3—C4—H4B	109.6 (10)	C20—C21—C22	120.24 (18)
H4A—C4—H4B	109.3 (14)	C20—C21—H21	119.0 (14)
C3A—C4A—C5A	121.45 (14)	C22—C21—H21	120.7 (14)
C3A—C4A—N3A	119.11 (15)	C21—C22—C23	120.70 (17)
C5A—C4A—N3A	119.27 (15)	C21—C22—H22	119.7 (11)
C5B—C4B—C3B	121.63 (13)	C23—C22—H22	119.6 (11)
C5B—C4B—N3B	119.06 (14)	C22—C23—C18	119.68 (15)
C3B—C4B—N3B	119.21 (14)	C22—C23—N3	121.32 (14)
N1—C5—C6	111.44 (12)	C18—C23—N3	118.92 (14)
			
O1—C1—C2—N1	−73.47 (17)	O6A—N1A—C6A—C5A	−30.2 (2)
C5—N1—C2—C1	−75.95 (15)	O7A—N1A—C6A—C5A	146.94 (16)
C3—N1—C2—C1	162.96 (13)	O6A—N1A—C6A—C1A	149.37 (18)
O3A—N2A—C2A—C3A	−16.0 (2)	O7A—N1A—C6A—C1A	−33.4 (2)
O2A—N2A—C2A—C3A	167.07 (13)	C4B—C5B—C6B—C1B	0.1 (2)
O3A—N2A—C2A—C1A	160.72 (14)	C4B—C5B—C6B—N1B	−177.32 (13)
O2A—N2A—C2A—C1A	−16.2 (2)	O1B—C1B—C6B—C5B	176.23 (16)
O1A—C1A—C2A—C3A	−172.63 (14)	C2B—C1B—C6B—C5B	−3.3 (2)
C6A—C1A—C2A—C3A	7.6 (2)	O1B—C1B—C6B—N1B	−6.4 (2)
O1A—C1A—C2A—N2A	10.9 (2)	C2B—C1B—C6B—N1B	174.07 (12)
C6A—C1A—C2A—N2A	−168.85 (12)	O6B—N1B—C6B—C5B	10.6 (2)
O1B—C1B—C2B—C3B	−174.15 (15)	O7B—N1B—C6B—C5B	−171.39 (14)
C6B—C1B—C2B—C3B	5.4 (2)	O6B—N1B—C6B—C1B	−166.93 (13)
O1B—C1B—C2B—N2B	4.6 (2)	O7B—N1B—C6B—C1B	11.1 (2)
C6B—C1B—C2B—N2B	−175.85 (12)	C4—N2—C7—C8	72.88 (16)
O2B—N2B—C2B—C3B	−127.14 (16)	C6—N2—C7—C8	−164.52 (12)
O3B—N2B—C2B—C3B	52.44 (19)	N2—C7—C8—C9	160.20 (12)
O2B—N2B—C2B—C1B	54.0 (2)	C10—N3—C9—C8	152.17 (13)
O3B—N2B—C2B—C1B	−126.42 (15)	C23—N3—C9—C8	−61.06 (17)
C5—N1—C3—C4	56.93 (14)	C7—C8—C9—N3	−56.46 (17)
C2—N1—C3—C4	178.96 (12)	C23—N3—C10—C11	−111.87 (16)
N2A—C2A—C3A—C4A	174.47 (13)	C9—N3—C10—C11	35.5 (2)
C1A—C2A—C3A—C4A	−2.1 (2)	C23—N3—C10—C15	71.41 (18)
C1B—C2B—C3B—C4B	−4.1 (2)	C9—N3—C10—C15	−141.22 (14)
N2B—C2B—C3B—C4B	177.17 (13)	C15—C10—C11—C12	2.4 (2)
C6—N2—C4—C3	58.56 (15)	N3—C10—C11—C12	−174.26 (14)
C7—N2—C4—C3	−178.06 (12)	C10—C11—C12—C13	0.6 (3)
N1—C3—C4—N2	−59.29 (15)	C11—C12—C13—C14	−1.8 (3)
C2A—C3A—C4A—C5A	−3.5 (2)	C12—C13—C14—C15	0.0 (3)
C2A—C3A—C4A—N3A	−178.66 (13)	C11—C10—C15—C14	−4.1 (2)
O4A—N3A—C4A—C3A	−1.6 (2)	N3—C10—C15—C14	172.67 (14)
O5A—N3A—C4A—C3A	177.50 (14)	C11—C10—C15—C16	174.51 (15)
O4A—N3A—C4A—C5A	−176.91 (15)	N3—C10—C15—C16	−8.7 (2)
O5A—N3A—C4A—C5A	2.2 (2)	C13—C14—C15—C10	2.9 (2)
C2B—C3B—C4B—C5B	0.2 (2)	C13—C14—C15—C16	−175.71 (16)
C2B—C3B—C4B—N3B	−176.01 (13)	C10—C15—C16—C17	−31.2 (3)
O5B—N3B—C4B—C5B	−9.7 (2)	C14—C15—C16—C17	147.44 (18)
O4B—N3B—C4B—C5B	171.44 (14)	C15—C16—C17—C18	3.0 (3)
O5B—N3B—C4B—C3B	166.71 (14)	C16—C17—C18—C23	30.6 (3)
O4B—N3B—C4B—C3B	−12.2 (2)	C16—C17—C18—C19	−150.09 (18)
C3—N1—C5—C6	−56.04 (15)	C23—C18—C19—C20	−0.2 (2)
C2—N1—C5—C6	−177.86 (12)	C17—C18—C19—C20	−179.58 (16)
C3A—C4A—C5A—C6A	2.6 (2)	C18—C19—C20—C21	0.5 (3)
N3A—C4A—C5A—C6A	177.78 (14)	C19—C20—C21—C22	−0.5 (3)
C3B—C4B—C5B—C6B	1.6 (2)	C20—C21—C22—C23	0.3 (2)
N3B—C4B—C5B—C6B	177.86 (13)	C21—C22—C23—C18	−0.1 (2)
C4—N2—C6—C5	−57.85 (15)	C21—C22—C23—N3	176.53 (14)
C7—N2—C6—C5	177.35 (12)	C19—C18—C23—C22	0.0 (2)
N1—C5—C6—N2	57.52 (15)	C17—C18—C23—C22	179.34 (15)
C4A—C5A—C6A—C1A	4.0 (2)	C19—C18—C23—N3	−176.67 (13)
C4A—C5A—C6A—N1A	−176.42 (14)	C17—C18—C23—N3	2.7 (2)
O1A—C1A—C6A—C5A	171.60 (15)	C10—N3—C23—C22	116.62 (16)
C2A—C1A—C6A—C5A	−8.7 (2)	C9—N3—C23—C22	−30.4 (2)
O1A—C1A—C6A—N1A	−8.0 (2)	C10—N3—C23—C18	−66.76 (18)
C2A—C1A—C6A—N1A	171.77 (13)	C9—N3—C23—C18	146.20 (14)

### Hydrogen-bond geometry (Å, °)

**Table:**

*D*—H···*A*	*D*—H	H···*A*	*D*···*A*	*D*—H···*A*
N1—H1···O1B^i^	0.91 (2)	1.85 (2)	2.6901 (16)	152.6 (18)
N1—H1···O7B^i^	0.91 (2)	2.383 (19)	3.0466 (17)	130.0 (16)
N2—H2···O1A^ii^	0.90 (2)	1.78 (2)	2.6204 (16)	154.6 (19)
N2—H2···O2A^ii^	0.90 (2)	2.43 (2)	3.0711 (16)	128.2 (16)
O1—H1C···O1B^i^	0.82	2.50	3.1600 (19)	138
O1—H1C···O7B^i^	0.82	2.38	3.0841 (18)	144
C2—H2A···O5B^ii^	0.94 (2)	2.43 (2)	3.3130 (19)	155.7 (16)
C2—H2A···O3B^iii^	0.94 (2)	2.60 (2)	3.2350 (19)	125.4 (15)
C3—H3B···O1^iv^	0.956 (18)	2.410 (18)	3.3250 (18)	160.0 (14)
C3B—H3D···O3B^v^	0.944 (19)	2.503 (19)	3.318 (2)	144.7 (15)
C4—H4B···O2A^ii^	0.970 (18)	2.648 (17)	3.1064 (18)	109.3 (12)
C5—H5A···O2A^vi^	0.947 (19)	2.418 (19)	3.2683 (18)	149.3 (14)
C5—H5B···O1A^ii^	0.988 (18)	2.523 (18)	3.1842 (18)	124.1 (13)
C5—H5B···O5B^ii^	0.988 (18)	2.714 (18)	3.5844 (19)	147.2 (14)
C6—H6B···O5A^i^	0.968 (19)	2.492 (19)	3.3728 (19)	151.2 (15)
C7—H7A···O4A^i^	0.99 (2)	2.44 (2)	3.371 (2)	158.0 (16)
C8—H8A···O2A^ii^	0.989 (17)	2.521 (17)	3.2870 (19)	134.1 (13)
C14—H14···O6A^vii^	0.95 (2)	2.47 (2)	3.085 (2)	122.0 (15)

Symmetry codes: (i) *x*+1, *y*, *z*; (ii) *x*, *y*+1, *z*; (iii) −*x*+1, −*y*+1, −*z*+1; (iv) −*x*+2, −*y*+1, −*z*+1; (v) −*x*, −*y*, −*z*+1; (vi) *x*+1, *y*+1, *z*; (vii) −*x*+2, −*y*+1, −*z*.

### Table 2 *Y*—*X*···Cg π ring interactions (Å)

Cg3 and Cg9 are the centroids of the C10–C15 and C1A–C6A rings, respectively.

**Table:**

*Y*—*X*···*Cg*	X···*Cg*	Y···*Cg*	X···Perp
C1A-O1A···Cg3^i^	3.5674 (13)	3.6471 (17)	3.494
N3A-O4A···Cg9^ii^	3.8172 (17)	3.8173 (17)	-3.357
N3B-O4B···Cg9^iii^	3.4320 (15)	3.9391 (15)	3.288

Symmetry codes: (i) x, -1+y, z; (ii) x, y, z; (iii) 1-x, -y, 1-z.

### Table 3 Cg···Cg π stacking interactions (Å)

Cg2, Cg3 , Cg8 and Cg9 are the centroids of the C10–C15, C18–C23, C1A–C6A and C1B–C6B rings, respectively.

**Table:**

	*Cg*X···*Cg*Y	CgX···Perp	CgY···Perp
Cg2···Cg2^i^	3.8038 (11)	-3.5589 (7)	-3.5590 (7)
Cg3···Cg3^i^	3.7164 (10)	-3.6624 (7)	-3.6623 (7)
Cg8···Cg9^ii^	3.9558 (10)	-3.2475 (6)	3.3731 (6)
Cg9···Cg8^ii^	3.9557 (10)	3.3730 (6)	-3.2475 (6)

Symmetry codes: (i) 2-x, 1-y, -z; (ii) x, y, z.
